# Mental health disorders in colorectal cancer patients following postoperative complications: Results from a single tertiary centre

**DOI:** 10.1192/j.eurpsy.2026.10653

**Published:** 2026-07-31

**Authors:** E. Shaska, F. Mulita, E. Liolis, L. Tchabashvili, K. Tasios, P. Leventis, N. Kornaros, A. Antzoulas, D. Litsas, G.-I. Verras

**Affiliations:** 1 Head of Department of Emergency Psychiatry, “Ali Mihali” Psychiatric Hospital, Vlora, Albania; 2 Department of Surgery, University of Patras, Patras; 3 Department of Surgery, General Hospital of Aigio, Aigio; 4 Department of Oncology, University of Patras; 5 Department of Surgery, General Hospital of Aigio, Patras; 6 Department of Surgery, General Hospital of Lamia, Lamia, Greece

## Abstract

**Introduction:**

In 2020, colorectal cancer (CRC) accounted for over 1.9 million newly diagnosed cases, positioning it as the third most prevalent cancer globally. During the same period, CRC was responsible for approximately 0.9 million fatalities, rendering it the second leading cause of cancer-related mortality worldwide. Significant geographical disparities have been documented concerning the incidence, mortality rates, temporal trends, and projected future burden of colorectal cancer across diverse countries and regions. Concurrently, shifts in the age-specific incidence patterns of CRC have been observed, characterized by a rising incidence among younger individuals, particularly evident in developed nations. Effective colorectal cancer prevention strategies involve two primary approaches: the identification and mitigation of modifiable risk factors, particularly those related to lifestyle (e.g., alcohol consumption, smoking, obesity, and an unhealthy diet), and the enhancement of protective factors (e.g., regular physical activity, adherence to specific chemo preventive medications such as aspirin, and the adoption of a healthy dietary pattern). Colorectal cancer (CRC) screening, a form of secondary prevention, is recognized as an effective prophylactic measure within CRC control programs.

**Objectives:**

This study aims to evaluate the prevalence of psychiatric morbidities after colorectal surgical procedures, identify associated risk factors for their development, and determine whether improved access to psychological therapies would benefit this patient population.

**Methods:**

In our study, 402 patients were investigated prospectively for psychiatric diseases as a complication one year postoperatively.

**Results:**

In our recent cohort of 402 CRC patients, 77 (19.2%) developed at least one new psychiatric diagnosis during follow-up. The most common conditions were mood disorders (4.23%)—predominantly depressive disorders (4%)—and anxiety disorders (3%), followed by delirium and other cognitive disturbances (1.74%).

**Conclusions:**

In conclusion, mental health disorders constitute a pivotal yet insufficiently acknowledged aspect of postoperative care for patients with colorectal cancer (CRC), particularly among those experiencing complications. The integration of systematic psychiatric evaluation and prompt intervention into CRC treatment protocols is crucial for enhancing both oncological outcomes and comprehensive recovery. Mental health is recognized as a fundamental component of oncological patient care, with existing evidence indicating its impact on overall survival.

**Disclosure of Interest:**

None Declared

